# Assessment of the Genetic Diversity of *Echinococcus multilocularis* from Copro-Isolated Eggs

**DOI:** 10.3390/pathogens10101296

**Published:** 2021-10-08

**Authors:** Jenny Knapp, Abdou Malik Da Silva, Sandra Courquet, Laurence Millon

**Affiliations:** 1 National Reference Centre for Echinococcoses, Department of Parasitology-Mycology, University Hospital of Besançon, 25030 Besançon, France; sandra.courquet@univ-fcomte.fr (S.C.); lmillon@chu-besancon.fr (L.M.); 2 Laboratoire Chrono-Environnement, UMR CNRS 6249 Université Bourgogne-Franche-Comté, 16 Route de Gray, 25030 Besançon, France; abdou_malik.da_silva@univ-fcomte.fr

**Keywords:** *Echinococcus multilocularis*, Taeniidae egg, EmsB marker, molecular epidemiology, genetic diversity

## Abstract

The genetic diversity of the parasite *Echinococcus multilocularis*, the infectious agent of alveolar echinococcosis, is generally assessed on adult worms after fox necropsy. We aimed to investigate *E. multilocularis* polymorphism through the microsatellite EmsB marker using a noninvasive approach. We tested batches of isolated eggs (1, 5, and 10) from 19 carnivore fecal samples collected in a rural town located in a highly endemic area in France to determine the best strategy to adopt using a minimal quantity of parasite DNA while avoiding genetic profile overlapping in the analysis. Several molecular controls were performed to formally identify the Taeniidae eggs. In total, 112 egg batches were isolated and 102 EmsB electrophoregrams were obtained in duplicate. Quality sorting was performed through the Pearson correlation coefficient (r) between each EmsB duplicate. Forty-nine batches with r > 0.9 remained in the analysis, mainly 5- or 10-egg batches. Three EmsB profiles were emphasized by hierarchical clustering and matched those from human lesions and adult worms previously genotyped and collected in the same area. We show that the genetic diversity of the parasite can be assessed from isolated *E. multilocularis* eggs in a spatiotemporal context using a noninvasive approach.

## 1. Introduction

*Echinococcus multilocularis* is a cestode responsible for alveolar echinococcosis disease. This parasite involves carnivores and herbivores in its life cycle, the red fox *Vulpes vulpes* being the main definitive host in Europe, and rodent micro-mammals as intermediate hosts [1]. Domestic animals, such as dogs (*Canis lupus familiaris*) and cats (*Felis catus*), can harbor the parasite, to a lesser extent [2], but can constitute a true threat in the spreading and transmission of the pathogen. Humans are involved in the life cycle and constitute a dead-end host for the parasite in the absence of predation. The infection is due to the accidental ingestion of eggs released in canid feces. Moreover, the presence of *E. multilocularis* eggs in cat feces collected in the field was previously demonstrated [3,4]. AE develops mostly in the liver, mimicking cancer, and can be lethal in untreated or inadequately treated cases [5]. However, it is now well accepted that only 1% of people who ingest the parasite actually develop the disease [6].

In definitive hosts, the parasite attaches to the wall of the small intestine using its double hanging system (two rows of hooks and four muscular suckers on the scolex) [7]. The parasite body strobila consists mainly of proglottids. At a certain place they are hermaphroditic, and at that place reproduction can occur. The terminal proglottid contains fertilized eggs, which are hexacanth embryos surrounded by several layers, ensuring its resistance outside when released from the host with the feces. Oncospheres, which represent the first larval stage, are released from eggs through the action of proteolytic enzymes in the digestive system of intermediate hosts [7]. Outside, the eggs are highly resistant to environmental conditions, especially cold (remaining infectious after 240 days at −18 °C, killed after 48 h at −83 °C) but are sensitive to desiccation (killed after 3 h at 43 °C at 85–95% relative humidity) [8]. *Taenia* spp. eggs are microscopically indistinguishable from one another and molecular biology is required to determine the species [9]. 

The parasite is spread in the environment by carnivores from endemic to hitherto *E. multilocularis*-free areas and its emergence or re-emergence is the subject of several recent studies [10]. An assessment of the prevalence in red foxes can be performed through, for example, national epidemiological campaigns [11]. However, fine spatiotemporal studies on the dispersion of the parasite can only be performed through the use of molecular markers [12]. As the parasite is a hermaphroditic species, it shows low genetic diversity [13], especially in the nucleotide genome [14]. However, its geographical dispersion can be assessed from microsatellite analyses. This is true for the tandemly repeated microsatellite EmsB [15]. Approximately 40 copies of this microsatellite are present in the *E. multilocularis* genome (located on chromosome 5, GenBank accession number AY680860) [16] and have been used in a number of studies on intermediate and definitive hosts at different geographical levels [17,18,19,20,21,22,23,24,25,26], notably to better understand the origin of the parasite in Europe [27]. It was shown that, at a local scale, the strains detected with an EmsB profile are shared and circulate among intermediate and definitive hosts and can be monitored over several years [26]. 

However, the samples analyzed in the aforementioned studies were isolated from adult worms following the necropsy of foxes and worm counting techniques were used, such as the segmental sedimentation and counting technique (SSCT) [28], or they were isolated from metacestode samples from micro-mammals or other intermediate hosts, necropsied animals, or surgery for humans. Necropsy is clearly not an option for studies conducted on domestic animals or pets. The aim of the present study was (1) to develop a high-quality EmsB genotyping method that can be performed on *E. multilocularis* eggs isolated from carnivore fecal samples, and (2) to assess the feasibility of conducting a genetic diversity study on *E. multilocularis* from noninvasive environmental sampling. Moreover, we attempted to assess the extent to which domestic or peri-domestic cycles are linked to and maintained by sylvatic cycles through definitive hosts.

## 2. Results

### 2.1. Copro-Sample and Egg Isolation

The total copro-DNA extracts of the 19 carnivore fecal samples selected for the study were positive for *E. multilocularis* rrnL by quantitative PCR, with Cq values from 28.66 to 41.04 and a mean Cq of 34.88 (95% confidence interval (CI): 32.99–36.76). No PCR inhibitors were detected (mean Alea Cq: 34.21, CI: 34.62–33.80). After sucrose flotation, 112 batches of 1, 5, and 10 eggs and egg pellets were isolated from the available specimens, corresponding to a minimum of 602 isolated eggs by microscopy. For each fecal sample, a batch (5 or 10 eggs) was tested by multiplex Trachsel PCR [29], which confirmed the species of the parasite by the detection of a specific band for the PCR product following electrophoreses.

**Figure 1:**
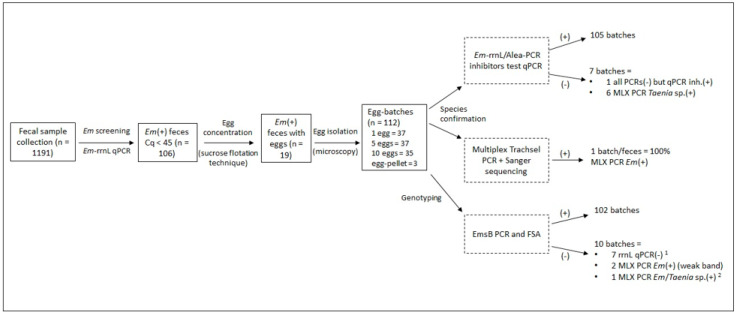
Results of *Echinococcus multilocularis* (*Em*) identification and EmsB typing on egg batches isolated from carnivore feces samples for which *E. multilocularis* DNA was detected. *E. multilocularis* identification was first performed using rrnL qPCR coupled to the Alea qPCR inhibitor test. Species confirmation was performed using the multiplex Trachsel PCR technique (MLX). *E. multilocularis* genotyping was performed by EmsB PCR followed by fragment size analysis (FSA). ^1^ batch (-) for all PCRs but Alea PCR(+); ^2^ batch *Em* rrnL qPCR(+).

The 112 egg batches were analyzed by rrnL qPCR for species confirmation. Detailed results are presented in Supplementary Data Table S1 and summarized in Figure 1. In total, 105 batches were positive (Cq value < 45 cycles). The seven remaining batches, originating from four fecal samples, were negative (Cq > 45 cycles). One of these batches was also negative by the multiplex Trachsel and EmsB PCRs. Thus, this sample probably did not contain *E. multilocularis* DNA. The six remaining batches were positive by multiplex Trachsel PCR, with a band specific for *Taenia* sp. that was confirmed by sequencing.

Genotyping using EmsB PCR was then performed in duplicate on all 112 batches and electrophoregrams for a fragment size analysis were obtained for 102. The 10 remaining batches, for which EmsB PCR gave no amplification, were isolated from seven fecal samples. Among them, seven were negative by *E. multilocularis* rrnL qPCR. *E. multilocularis* DNA was detected for the other three using the other PCR techniques. Among the EmsB-negative batches, six showed a *Taenia*-specific band (267 bp) following multiplex Trachsel PCR, two a weak *E. multilocularis*-specific band (395 bp) (data not shown), and one a double band (*E. multilocularis* and *Taenia* spp. bands). 

After a Sanger sequencing of the multiplex Trachsel PCR products with a specific *Taenia* spp. band, the presence of the parasites *Taenia crassiceps* (100% identity with the Genbank reference sequence MN505206.1) and *Taenia polyacantha* (100% identity with LT635753.1) were detected in two fecal samples each (Supplementary Data Table S1). 

### 2.2. EmsB Fragment Size Analysis and Comparison of Pearson Coefficient Sorting

All EmsB PCRs were performed in duplicate for each batch and the reproducibility of the electrophoregram patterns between the PCR duplicates was assessed through Pearson coefficient scores to ensure the quality of the genotyping.

**Figure 2:**
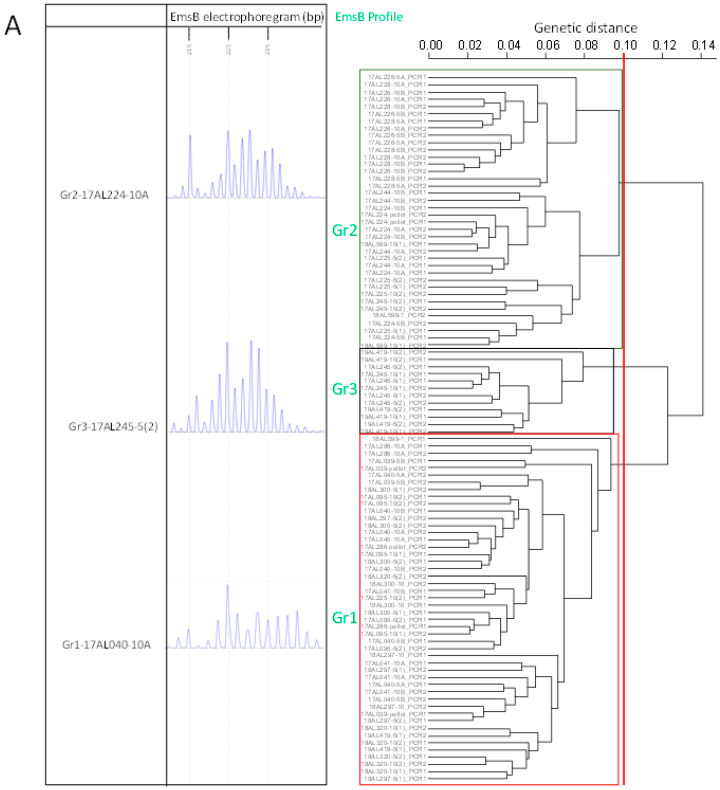

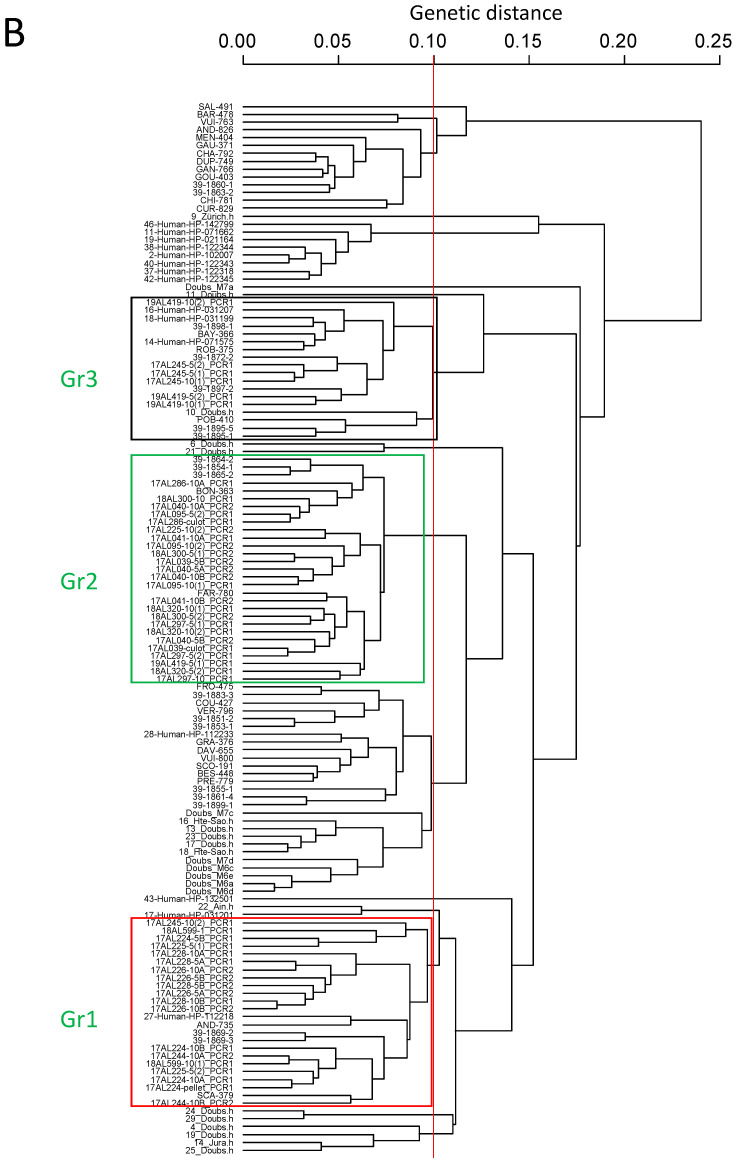
Dendrograms constructed from EmsB amplification data after hierarchical clustering analysis based on Euclidean distances and the unweighted pair group method. A genetic threshold of 0.1 was applied for EmsB profile discrimination. Representative electrophoregrams are provided for each profile. (**A**) Dendrogram composed of EmsB PCR pattern duplicates (PCR1, PCR2) on 49 egg batches from 17 carnivore feces samples with a Pearson correlation coefficient > 0.9. (**B**) Dendrogram composed of the 49 egg batches and previously genotyped human lesions (*n* = 60) and adult worms (*n* = 25).

Dendrograms were generated using all EmsB-positive batches (*n* = 102) and then with a selection of batches sorted as a function of the Pearson coefficient scores (r > 0.75, > 0.8, and > 0.9) (Figure S1). Most of the EmsB patterns fit into three main profiles (Gr1, Gr2, and Gr3) (Figure 2A). Other patterns were either unreadable or non-reproductible and are referred to as “out of the main profiles”. In the dendrogram that included all 102 batches, the patterns of the two duplicates were classified in the same profile for 61 (29 batches in Gr1, 25 batches in Gr2, and 6 in Gr3) (Supplementary Data Table S1 and Figure 2A). For 20 batches, only one electrophoregram pattern among the duplicates was clustered in one of the main profiles, the other one being out of the main profiles. For two batches, the patterns of each duplicate were clustered in two separate profiles and for 20, the patterns of the two duplicates were classified out of the main profiles.

For the dendrogram built with pattern duplicates showing r > 0.75 (*n* = 73 batches), 59 batches showed both patterns clustering in the same profile, eight with one pattern classified out of the main profiles, three with patterns classified in two different profiles, and three with both duplicate patterns classified out of the main profile. For r > 0.8 (68 batches), 58 batches showed both patterns clustered in the same profile, seven with one pattern classified out of the main profiles, two with patterns classified in two different profiles, and one with both duplicates classified out of the main profile. Finally, for r > 0.9 (49 batches), 47 batches showed both patterns in the same profile, two with the patterns classified in two different profiles, and none were classified out of the main profile (Table 1).

The batches sorted with r > 0.9 and the PCR duplicate patterns with the highest electrophoregram fluorescence intensity were used (*n* = 49 batches) for the hierarchical clustering analysis with AE lesions from French and Swiss patients (*n* = 60) and fox adult worms from the Doubs and Jura French *départements* (*n* = 25) of the study area. The three profiles found in this report clustered with the human lesions and adult worm samples (Figure 2B). The patients clustered with the egg batches were all from the Franche-Comté *Région* or neighboring Swiss cantons. The Gr1 profile was associated with the previously described P4/P5 profiles [26], two similar profiles found in humans, profile Gr2 with P1/P3, and profile Gr3 with P9, a cross-border profile found in humans.

### 2.3. Correlation between EmsB Profiles and Individual Identification of Carnivores

Eight different foxes and two dogs were identified from the 19 carnivore feces samples, with the number of copro-samples per individual ranging from one to three (Table 2). Only one fecal sample was attributed to a cat. Three fox and one dog fecal samples were not individually identified. Foxes showed the three EmsB profiles described from eggs, dogs the Gr1 and Gr3 profiles, and the cat the Gr1 profile.

## 3. Discussion

Genotyping of individual *E. multilocularis* eggs is challenging but required for several purposes. There is a strong need to improve this method using a noninvasive approach in the context of both laboratory animal studies and wild and domestic animal surveys [30]. Indeed, research can be conducted without sacrificing animals in both cases. Moreover, noninvasive approaches are required for *E. multilocularis* circulation studies among animals in the environment and the follow-up of contamination and re-contamination of individuals. Until now, EmsB genotyping of *E. multilocularis* specimens in animals has been conducted on isolated adult worms after necropsy, either of definitive or intermediate hosts [22,24,27,31,32], or from lesion resections [25,26]. The amount of DNA was not limiting in these contexts. For genotyping performed on isolated eggs, only small quantities of DNA are available, but EmsB PCR is theoretically possible on 1 fg of *E. multilocularis* DNA [15]. Indeed, a single egg was estimated to contain 8 pg of nuclear DNA [33] and certainly more considering the total genomic and mitochondrial DNA. However, EmsB genotyping on *E. multilocularis* eggs isolated from environmental samples had thus far never been performed and needed to be assessed.

The first challenge was to isolate the specimens of *E. multilocularis* eggs from carnivores potentially infected by several Taeniidae species. A microscopic examination allows identification at the Taeniidae family level. Species identification is only possible by molecular diagnosis. From among the 19 carnivore feces samples positive by rrnL qPCR [34] performed on the total DNA extract and positive for the presence of *E. multilocularis* eggs, one fecal sample was found to contain mostly other Taeniidae eggs. This was identified by sequencing as *Taenia crassiceps*, a common tapeworm in the red fox [35], and no amplification of the EmsB target from the studied egg batches was observed for several batches. However, *E. multilocularis* eggs were certainly present in the stool sample but represented a minority, as EmsB amplification was possible for one batch. For individual batches, the combination of the rrnL qPCR and EmsB PCR assays allowed for the efficient identification of *E. multilocularis*. Species confirmation was reinforced by multiplex Trachsel PCR [29]. The specificity of the rrnL qPCR assay was previously tested [34] and confirmed here. However, multi-infection with different *Taenia* spp. and *E. multilocularis* could not be detected by this technique. A combination of three PCR assays (rrnL qPCR, EmsB, and the multiplex Trachsel PCR) is thus required for formal identification.

The second challenge was the low quantity of available DNA, especially for the one-egg batches. EmsB PCR was thus performed in duplicate to control the reproducibility of the results. Using the dendrogram built from all of the available electrophoregrams (from 102 batches), 19.6% of the PCR duplicates (20/102 batches) were not classified in any of the three main profiles, of which 70% came from one-egg batches. Quality sorting by the Pearson correlation coefficient between EmsB pattern duplicates allowed us to select batches with reproducible results that could be retained for the genetic analysis. Considering at least one pattern duplicate classified in a profile, for r > 0.75, 95.9% of the samples (70/73 batches) were classified among Gr1, Gr2, or Gr3, for r > 0.8, 98.5% of the samples (67/68 batches) were classified, and finally 100% of the samples were classified for r > 0.9. However, considering the two pattern duplicates, for r > 0.75, 80% of the samples (59/73 batches) were classified, for r > 0.8, 85.3% of the samples (58/68 batches) were classified, and 100% of the samples were classified for r > 0.9. This result emphasizes the necessity to control the reproducibility of EmsB electrophoregrams by duplicate PCR combined with Pearson coefficient-based sorting. However, it appears to be difficult to use one-egg batches in the analysis with this process. After r > 0.75 sorting, only 10/37 one-egg batches remained in the analysis, with two having a PCR duplicate correctly classified in the same profile. Reproducibility of the EmsB PCR was more efficient with 5- to 10-egg batches. Thus, the genotype analysis on a single egg requires the analysis of more one-egg batches or fresher samples isolated by sucrose flotation more rapidly after sampling. Indeed, a time lapse of one to three years was observed between sampling and the EmsB amplification, likely compromising the results.

Nonetheless, most fecal samples showed a unique profile among the eggs of the same batch, except for three, which showed EmsB pattern duplicates clustered in different profiles. This could reflect the presence of rare mixed infections among foxes and dogs, exposing them to possible multi-infections through intense predation [27]. Even if foxes and dogs are infected with several different EmsB genotypes, the non-reproducibility of the results could be detected among batches by our procedure. The genotyping of adult worms in a European study showed several genotypes in the same fox [27]. Foxes harbored several different *E. multilocularis* populations of worms, arising from different events of predation and the worms were certainly at different maturation stages at the necropsy examination but counted together, e.g., an SSCT assay. The fact that no overlapping profiles on electrophoregrams were observed to be “hybrid profiles” could be due to the analysis of single *E. multilocularis* egg populations, sequentially released in the feces at the moment. This result makes it possible to consider EmsB genotyping of *E. multilocularis* from 5- to 10-egg batches with confidence. 

We then assessed the diversity of the parasite within a limited geographical area (3 km²), emphasizing the presence of three EmsB profiles among the animals. Wild and domestic animals appear to share two of the three described profiles, the three dogs and the cat sharing only Gr1 and Gr3 with the foxes, whereas the foxes harbored the three profiles. Foxes probably transmitted *E. multilocularis* to domestic animals and not the contrary; however, further studies need to be performed to confirm this hypothesis. Moreover, individual identification of animals was possible for some of them. Two foxes were the origins of several stools and showed one profile each. Further investigations must be carried out in this direction to assess the spatiotemporal spreading of the parasite among carnivore hosts. 

The combination of EmsB results obtained on egg batches, adult worms, and human lesions confirmed the description of the three profiles. The EmsB profiles from egg batches matched those from parasites collected from foxes or patients from the same region or the neighboring country, Switzerland, thus confirming the local circulation of these three profiles among hosts.

## 4. Materials and Methods

**Figure 3:**
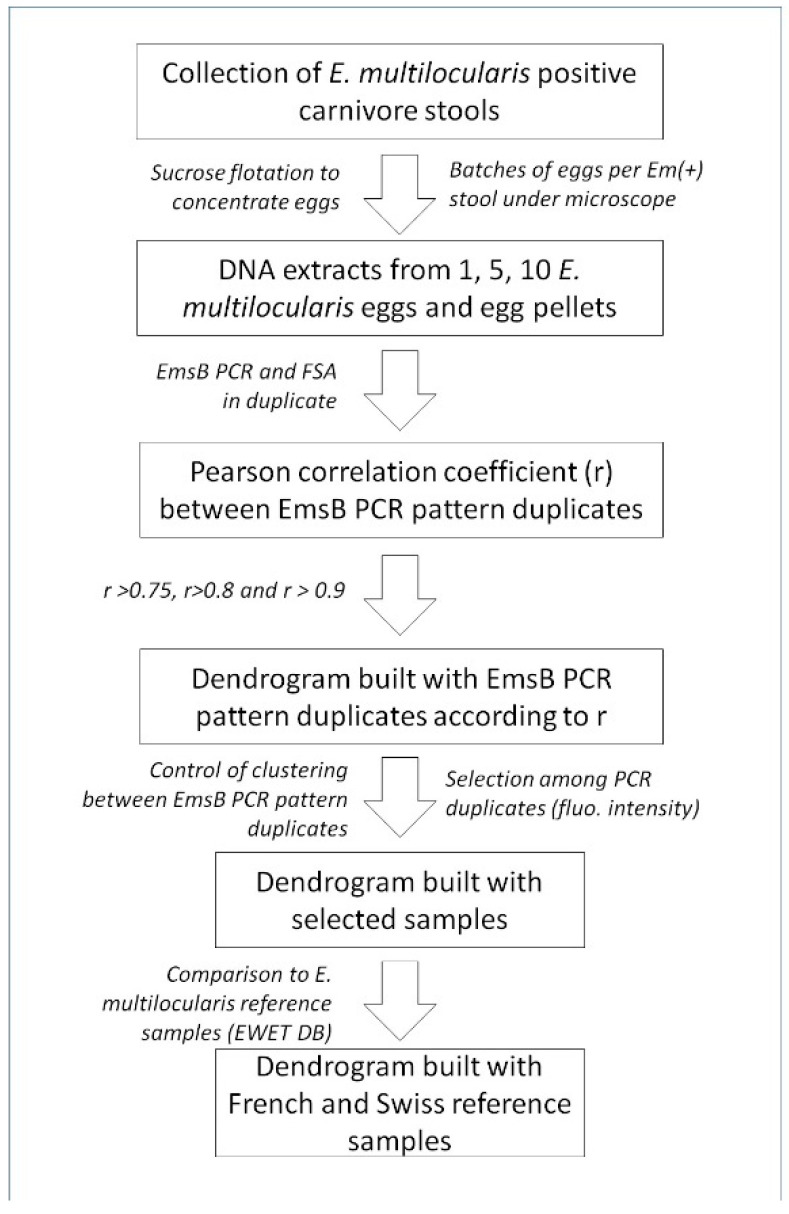
Cynoptic diagram for the experimental approach of the present study. Em(+): positive *Echinococcus multilcularis* fecal samples, FSA: fragment size analysis, EWET DB: EmsB Website for *Echinococcus* Typing Database.

The experimental approach of the present study is summarized in Figure 3. 

### 4.1. Fecal Samples, Egg Isolation, and DNA Purification

From a collection of carnivores fecal samples (*n* = 1191) [3], 106 were found to be positive for *E. multilocularis* by rrnL quantitative PCR (qPCR) on DNA stool extracts (QIAmp Fast DNA Stool Minikit, Qiagen, Hilden, Germany) and 19 were selected for egg isolation [3]. They were sampled in a rural settlement in the Eastern part of France, in a historically endemic area. The identity of the host was determined by a host fecal qPCR test, and the presence of *E. multilcularis* DNA and the absence of PCR inhibitors were checked [34]. Briefly, the targeted DNA (*E. multilocularis* DNA, host DNA, and internal control plasmid) was detected by a 45-cycle qPCR in the presence of specific Taqman probes. The presence of *E. multilocularis* DNA was determined by a cycle threshold (Cq) < 45, host DNA by a Cq < 26, and the internal control Alea by a Cq < 37 [34]. The individual genetic identity of the host was determined by performing a microsatellite analysis, based on 14 microsatellite markers specific for the red fox (Da Silva et al. in prep) and 22 microsatellite markers for the dog (Knapp et al. in prep.), with one gender target applied for the two carnivores. Cat feces were not individually identified.

Based on the results of the *E. multilocularis* qPCR assays, a sucrose flotation technique was performed, as described in Matsuo and Kimiya (2005) and Escotte-Binet et al. (2019) [36,37]. This method, combined with microscopic observation and qPCR, had a sensitivity of 100% for the detection of 10 eggs in 5 g of feces and the potential recovery of only one egg in 5 g of feces [3]. Briefly, eggs were concentrated and isolated based on the differential specific gravity (SG) between the eggs and particles. Five grams of carnivore feces were dispersed in 20 mL 0.1% Tween 80. Eggs contained in the floating pellets were separated from the organic particles. The flotation mixture was centrifuged at 2500× *g* for 10 min and the pellet was resuspended and deposited on a sucrose solution with SG = 1.27. After centrifugation, the supernatants containing the eggs were concentrated in <1 mL volume. Taeniid eggs were isolated using a Leica DMI3000-B micromanipulator (Leica Microsystems, Wetzlar, Germany), equipped with an inverted microscope to identify Taeniid eggs at a 400× magnification. Depending on the quantity of available Taeniid eggs from the flotation pellets, batches were constituted with 1, 5, or 10 eggs or egg pellets (>10 eggs). The DNA from egg batches was purified using the QIAmp DNA Mini Kit (Qiagen, Hilden, Germany) after overnight digestion, following the manufacturer’s recommendations, and finally eluted in a volume of 50 µL of the provided buffer. Each egg batch was tested for the presence of *E. multilocularis* DNA by *E. multilocularis*-specific qPCR (part of the mitochondrial *rrnL* gene), as well as the presence of PCR inhibitors with the Alea internal control test [34]. One additional PCR was performed for one batch per stool sample, for *E. multilocularis* confirmation or in case of non-amplification of the *rrnL* gene part or EmsB microsatellite, using the multiplex PCR developed by Trachsel et al. [29], which distinguishes between *E. multilocularis* (part of the *nad1* gene, 395 bp, GenBank accession number AB018440), *E. granulosus* (part of the *rrnS* gene, 117 bp, GenBank accession number AF297617), and *Taenia* spp. (part of the *rrnS* gene, 267 bp). Multiplex PCR amplicons were visualized by electrophoresis, in which 8 µL of the PCR product was loaded onto 1.5% (*w/v*) agarose gels, stained with 10% SYBR Safe DNA Gel Stain (Invitrogen, Carlsbad, CA, USA) in 1 X tris-acetate EDTA solution, run for 30 min at 100 V, and viewed under UV light using a Gel Logic 100 Imaging System and Scientific Imaging System v3.6.1 software (Kodak, New Haven, CT, USA). The confirmation of the species identity for *Taenia* spp.-specific bands visualized following the multiplex PCR was performed by Sanger sequencing of the PCR products using the Ces5seq primer [29].

### 4.2. EmsB Amplification and Fragment Size Analyses

The EmsB PCR reaction was performed in an 18 µL final volume with a multiplex PCR master mix containing 2.8 U HotStarTaq DNA polymerase (Qiagen, Hilden, Germany), 0.1 µM final concentration of each primer (EmsB A: 5′-FAM-GTGTGGATGAGTGTGCCATC-3′, EmsB C: 5′-CCACCTTCCCTACTG-CAATC-3′), 3% DMSO, and up to 1 µL of purified DNA. PCR was performed in a Biometra T3 thermocycler (Whatman Biometra, Goettingen, Germany), as described by Knapp et al. [26]. For each run, a plasmid construction, the EmsB calibrator [38], was used to allow comparison of the fragment size [25]. Four EmsB patterns are included in the plasmid, corresponding to (CA)_9–11_ (GA)_11_ repeats, two microsatellites of the same size of 190 bp, the second of 192 bp, and the last of 194 bp [38]. Each DNA extract was amplified in duplicate.

The fragment size analyses were performed from the dyed PCR products by capillary electrophoresis on the SeqStudio genetic analyzer device (Applied Biosystems, Foster City, CA). Electrophoregrams, obtained from fragment size analyses, were analyzed online using the microsatellite analysis application available on the ThermoFisher Scientific website.

### 4.3. Hierarchical Clustering Analysis and Pearson Coefficient Sorting

The peaks present on the electrophoregrams and their heights, based on fluorescence intensity, were analyzed. These two parameters were used to (i) compare the quality of the genotype made for each duplicate sample, and (ii) study the genetic diversity of the parasite in the study area. Hierarchical clustering was performed using the Euclidean distance and the unweighted pair group method with arithmetic mean (UPGMA) [23,39] was applied to all tested batches or after sorting based on the Pearson correlation coefficient. RStudio software (R version 3.5.1) [40] was used to perform the clustering analyses using the package pvclust [41]. A genetic distance threshold of 0.1 was applied; under this threshold two samples were considered to have an identical EmsB profile [26]. Pearson correlation coefficients (r) were determined to compare the quality of the duplicate PCR for each sample [23] and different levels were applied to determine the relevance of the quality of the EmsB PCR duplicates (selection of duplicates with r > 0.75, > 0.8, > 0.9, or the whole batch considered and used to build the dendrograms). Genetic diversity among the samples was assessed by determining the EmsB profiles using three steps to build the dendrogram. First, PCR duplicate couples were applied to the dendrogram. A duplicate PCR had to be grouped in the same profile to be validated. Second, for a given sample, the PCR duplicate with the higher fluorescence intensity sum was retained. Third, the selected egg batches from the present study were compared to French and Swiss *E. multilocularis* samples genotyped and publicly available on the EmsB website for the *Echinococcus* Typing (EWET) project, by adding them to the dendrogram [38]. 

### 4.4. Correlation between EmsB Profiles and Individual Identification of Carnivores

EmsB profiles harbored by individually identified animals were studied by data comparison (Table 2).

## 5. Conclusions

This study tested the EmsB marker on a new matrix: isolated eggs from noninvasive sampling. A protocol was then implemented to ensure the relevance of the results by specific PCR controls and an EmsB analysis in duplicate. The use of 5 to 10-egg batches was found to be sufficient to study the genetic diversity of *E. multilocularis* in the environment using a spatiotemporal approach. Investigations on larger geographical areas could be conducted using this procedure to assess parasite spreading among wild and domestic animals on larger studied endemic regions.

## Figures and Tables

**Table 1:** Egg-batch genotyping after dendrogram construction and Pearson correlation coefficient (r) sorting. Gr1, Gr2, and Gr3 correspond to profiles in which the EmsB PCR pattern duplicates clustered together in a profile. Gr1/Gr2 or Gr1/Gr3 correspond to EmsB PCR pattern duplicates classified in separate profiles. P: egg pellet, OT: samples out of threshold, /: samples removed from the analysis after r sorting, NE: batches not available.

	Full Batches							r > 0.75							r > 0.8							r > 0.9						
Faeces Code	1	1	5	5	10	10	p	1	1	5	5	10	10	p	1	1	5	5	10	10	p	1	1	5	5	10	10	p
17AL039	OT	OT	G1 ^1^	G1	G1	G1	G1	/	/	/	G1	G1	G1	G1	/	/	/	G1	G2	G3	G1	/	/	/	G1	/	/	G1
17AL040	OT	G3 ^1^	G1	G1	G1	G1	-	/	/	G1	G1	G1	G1	-	/	/	G1	G1	G1	G1	-	/	/	G1	G1	G1	G1	-
17AL041	G1 ^1^	G1	G1	G1	G1	G1	-	G1 ^1^	G1	G1	G1	G1	G1	-	G1 ^1^	/	G1	G1	G1	G1	-	/	/	/	/	G1	G1	-
17AL095	NE	OT	G1	G1	G1	G1	-	NE	/	G1	G1	G1	G1	-	NE	/	G1	G1	G1	G1	-	NE	/	/	G1	G1	G1	-
17AL224	G2 ^1^	G2 ^1^	G2 ^1^	G2	G2	G2	G2	/	G2 ^1^	G2 ^1^	G2	G2	G2	G2	/	G2 ^1^	G2 ^1^	G2	G2	G2	G2	/	/	/	G2	G2	G2	G2
17AL225	OT	NE	G2	G2	G2	G1/G2	-	OT	NE	G2	G2	G2	G1/G2	-	OT	NE	G2	G2	G2	G1/G2	-	/	NE	G2	G2	/	G1/G2	-
17AL226	G2 ^1^	OT	G2	G2	G2	G2	-	G2 ^1^	/	G2	G2	G2	G2	-	G2 ^1^	/	G2	G2	G2	G2	-	/	/	G2	G2	G2	G2	-
17AL228	G2 ^1^	G2 ^1^	G2	G2	G2	G2	-	/	G2 ^1^	G2	G2	G2	G2	-	/	/	G2	G2	G2	G2	-	/	/	G2	G2	G2	G2	-
17AL244	OT	NE	G2	G2	G2	G2	-	/	NE	G2	G2	G2	G2	-	/	NE	G2	G2	G2	G2	-	/	NE	/	/	G2	G2	-
17AL245	NE	G2 ^1^	G3	G3	G3	G2	-	NE	G2 ^1^	G3	G3	G3	G2	-	NE	G2 ^1^	G3	G3	G3	G2	-	NE	/	G3	G3	G3	G2	-
17AL286	G1 ^1^	NE	OT	G1 ^1^	G1	G1 ^1^	G1	/	NE	/	/	G1	OT	G1	/	NE	/	/	G1	/	G1	/	NE	/	/	G1	/	G1
18AL297	G2	OT	G1	G1	G1	-	-	/	/	G1	G1	G1	-	-	/	/	G1	G1	G1	-	-	/	/	G1	G1	G1	-	-
18AL300	G1 ^1^	OT	G1	G1	G1	-	-	/	/	G1	G1	G1	-	-	/	/	G1	G1	G1	-	-	/	/	G1	G1	G1	-	-
18AL320	G1 ^1^	NE	G1 ^1^	G1	G1	G1	-	OT	NE	G1/G3	G1	G1	G1	-	/	NE	/	G1	G1	G1	-	/	NE	/	G1	G1	G1	-
18AL344	OT	OT	G2 ^1^	OT	OT	G1 ^1^	-	/	/	G1 ^1^	/	/	/	-	/	/	G1 ^1^	/	/	/	-	/	/	/	/	/	/	-
18AL460	NE	-	NE	OT	NE	NE	-	NE	-	NE	/	NE	NE	-	NE	-	NE	/	NE	NE	-	NE	-	NE	/	NE	NE	-
18AL599	G2	OT	G2	G1/G2	G2	G2	-	G2	/	G2	G1/G2	G2	G2	-	G2	/	G2	G1/G2	G2	G2	-	G1/G2	/	/	/	G2	/	-
19AL127	OT	OT	OT	-	OT	-	-	/	/	/	-	/	-	-	/	/	/	-	/	-	-	/	/	/	-	/	-	-
19AL419	G1 ^1^	G3 ^1^	G1	G3	G3	G3	-	/	G3 ^1^	G1	G3	G3	G3	-	/	G3 ^1^	G1	G3	G3	G3	-	/	/	G1	G3	G3	G3	-

^1^ EmsB PCR pattern duplicates for which only one fragment size analysis was classified in a profile, the other being classified out.

**Table 2:** Collection of 19 carnivore feces samples, details on sampling and flotation, and molecular identification of the host and the parasite. *E. multilocularis*: *Echinococcus multilocularis. Canis l. familiaris:* *Canis lupus familiaris.*

				qPCR Cq Value ^1^				No of Batches Per Egg-Batch Type				
Feces Code	Host ID	Molecular Host	Sampling Date	Host	Em rrnL	PCR Inhib. Test	Batch Species Typing ^2^	1	5	10	Pellet	Total
17AL039	FOX005	*Vulpes vulpes*	July 2017	25.85	28.66	32.92	*E. multilocularis*	2	2	2	1	7
17AL040	FOX005	*Vulpes vulpes*	July 2017	22.20	31.24	33.86	*E. multilocularis*	2	2	2	0	6
17AL041	FOX005	*Vulpes vulpes*	July 2017	22.62	30.08	33.49	*E. multilocularis*	2	2	2	0	6
17AL095	DOG001	*Canis l. familiaris*	March 2017	21.31	41.04	35.63	*E. multilocularis*	2	2	2	0	6
17AL224	FOX004	*Vulpes vulpes*	May 2017	24.30	33.76	34.99	*E. multilocularis*	2	2	2	1	7
17AL225	FOX020	*Vulpes vulpes*	May 2017	23.70	37.59	35.53	*E. multilocularis*	2	2	2	0	6
17AL226	ND	*Vulpes vulpes*	May 2017	27.53	31.44	34.55	*E. multilocularis*	2	2	2	0	6
17AL228	ND	*Vulpes vulpes*	May 2017	32.25	33.14	35.37	*E. multilocularis*	2	2	2	0	6
17AL244	FOX004	*Vulpes vulpes*	May 2017	24.64	36.50	34.90	*E. multilocularis*	2	2	2	0	6
17AL245	FOX017	*Vulpes vulpes*	May 2017	27.91	39.58	34.81	*E. multilocularis*	2	2	2	0	6
17AL286	CAT001	*Felis catus*	July 2017	21.57	38.61	33.92	*E. multilocularis*	2	2	2	1	7
18AL297	ND	*Vulpes vulpes*	May 2018	26.79	37.94	33.56	*E. multilocularis*	2	2	1	0	5
18AL300	DOG010	*Canis l. familiaris*	July 2018	21.75	38.52	33.71	*E. multilocularis*	2	2	1	0	5
18AL320	FOX031	*Vulpes vulpes*	November 2018	23.09	38.97	33.11	*E. multilocularis*	2	2	2	0	6
18AL344	FOX031	*Vulpes vulpes*	November 2018	30.07	37.51	33.22	*E. multilocularis*	2	2	2	0	6
18AL460	FOX032	*Vulpes vulpes*	September 2018	22.82	31.64	34.59	*Taenia crassiceps*	1	2	2	0	5
18AL599	FOX039	*Vulpes vulpes*	November 2018	23.39	30.09	34.05	*E. multilocularis*	2	2	2	0	6
19AL127	FOX030	*Vulpes vulpes*	April 2019	27.34	35.72	34.46	*E. multilocularis*	2	1	1	0	4
19AL419	ND	*Canis l. familiaris*	November 2019	21.06	30.59	33.41	*E. multilocularis*	2	2	2	0	6
							Total No of batches	37	37	35	3	112

^1^ qPCR performed on the total DNA extract from carnivore stool samples (qPCR performed in duplicate, the average Cq is reported). ^2^ Typing performed by the multiplex PCR designed by Trachsel et al. [29].

## Supplementary Materials

The following are available online at https://www.mdpi.com/article/10.3390/pathogens10101296/s1, Figure S1. Dendrograms constructed using (a) all available EmsB PCR duplicates (102 batches) and after Pearson correlation coefficient sorting (B to D), Table S1. Egg batch collection, molecular identification, and EmsB genotyping depending on the Pearson correlation coefficient (r) sorting. Gr1, Gr2, and Gr3 correspond to EmsB duplicates clustered in the same profile. Profiles Gr1/Gr2 or Gr1/Gr3 correspond to EmsB PCR duplicates classified in separate profiles. OT: samples out of threshold. /: samples removed from the analysis after r sorting.
