# Supplementary material for: Assessment of the Genetic Diversity of Echinococcus multilocularis from Copro-Isolated Eggs
Source: Pathogens. 2021 Oct 8;10(10):1296. doi: 10.3390/pathogens10101296 (PMC8541330; doi:10.3390/pathogens10101296)
Supplement: Supplementary file 1 [file pathogens-10-01296-s001.zip › Supplementary data Figure 1.pdf]

(a)

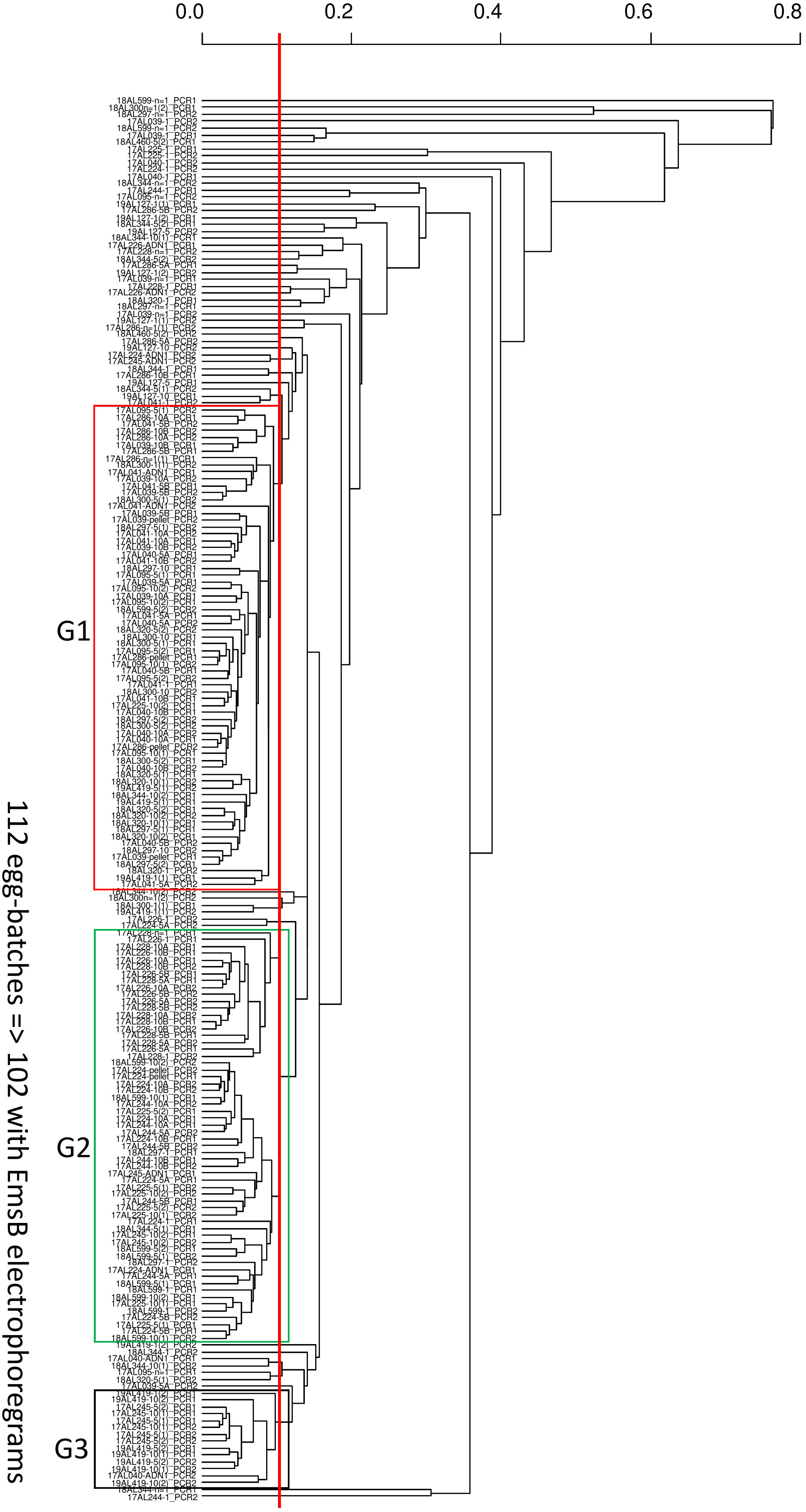
0.0
0.2
0.4
0.6
0.8
G1
G2
G3
112 egg-batches => 102 with EmsB electrophoregrams

(b)

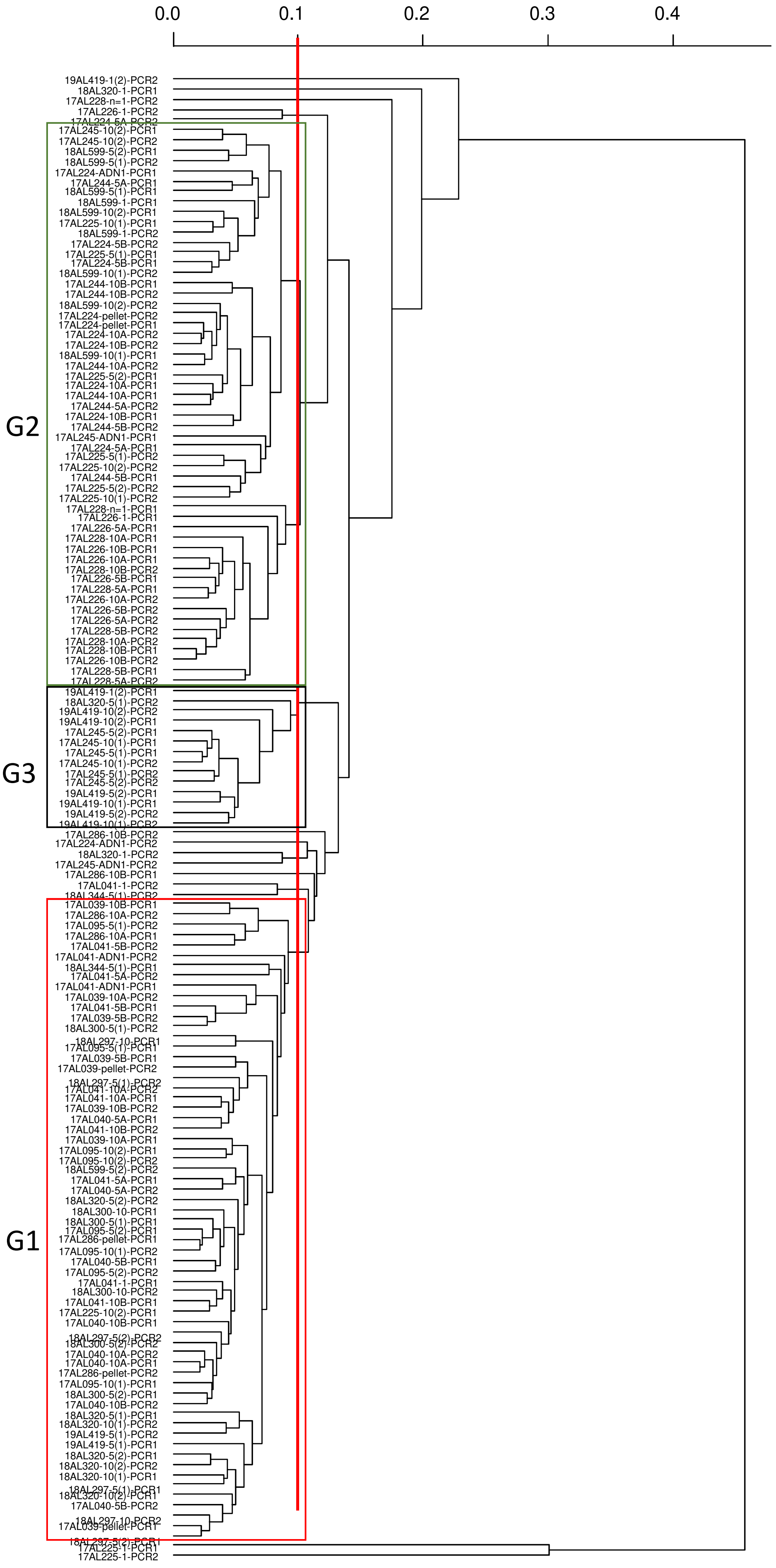

Pearson correlation coefficient = r > 0.75 – 73 egg-batches

(c)

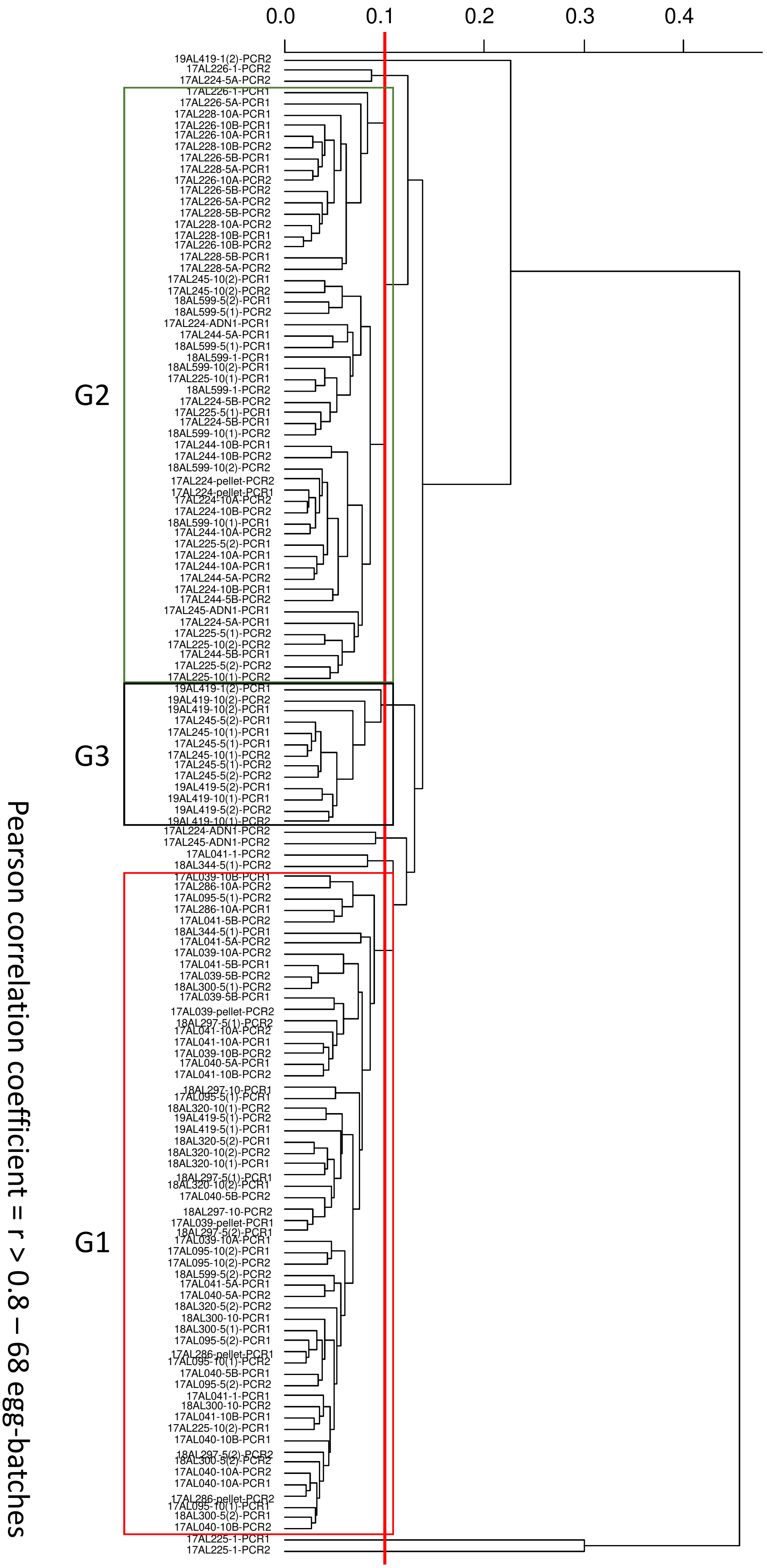

0.0
0.1
0.2
0.3
0.4
G2
G3
G1
Pearson correlation coefficient = r > 0.8 – 68 egg-batches
19AL419-1(2)-PCR2
17AL226-1-PCR2
17AL224-5A-PCR2
17AL226-1-PCR1
17AL226-5A-PCR1
17AL228-10A-PCR1
17AL226-10B-PCR1
17AL226-10A-PCR1
17AL228-10B-PCR2
17AL226-5B-PCR1
17AL228-5A-PCR1
17AL226-10A-PCR2
17AL226-5B-PCR2
17AL226-5A-PCR2
17AL228-5B-PCR2
17AL228-10A-PCR2
17AL228-10B-PCR1
17AL226-10B-PCR2
17AL228-5B-PCR1
17AL228-5A-PCR2
17AL245-10(2)-PCR1
17AL245-10(2)-PCR2
18AL599-5(2)-PCR1
18AL599-5(1)-PCR2
17AL224-ADN1-PCR1
17AL244-5A-PCR1
18AL599-5(1)-PCR1
18AL599-1-PCR1
18AL599-10(2)-PCR1
17AL225-10(1)-PCR1
18AL599-1-PCR2
17AL224-5B-PCR2
17AL225-5(1)-PCR1
17AL224-5B-PCR1
18AL599-10(1)-PCR2
17AL244-10B-PCR1
17AL244-10B-PCR2
18AL599-10(2)-PCR2
17AL224-pellet-PCR2
17AL224-pellet-PCR1
17AL224-10A-PCR2
17AL224-10B-PCR2
18AL599-10(1)-PCR1
17AL244-10A-PCR2
17AL225-5(2)-PCR1
17AL224-10A-PCR1
17AL244-10A-PCR1
17AL244-5A-PCR2
17AL224-10B-PCR1
17AL244-5B-PCR2
17AL245-ADN1-PCR1
17AL224-5A-PCR1
17AL225-5(1)-PCR2
17AL225-10(2)-PCR2
17AL244-5B-PCR1
17AL225-5(2)-PCR2
17AL225-10(1)-PCR2
19AL419-1(2)-PCR1
19AL419-10(2)-PCR2
19AL419-10(2)-PCR1
17AL245-5(2)-PCR1
17AL245-10(1)-PCR1
17AL245-5(1)-PCR1
17AL245-10(1)-PCR2
17AL245-5(1)-PCR2
17AL245-5(2)-PCR2
19AL419-5(2)-PCR1
19AL419-10(1)-PCR1
19AL419-5(2)-PCR2
19AL419-10(1)-PCR2
17AL224-ADN1-PCR2
17AL245-ADN1-PCR2
17AL041-1-PCR2
18AL344-5(1)-PCR2
17AL039-10B-PCR1
17AL286-10A-PCR2
17AL095-5(1)-PCR2
17AL286-10A-PCR1
17AL041-5B-PCR2
18AL344-5(1)-PCR1
17AL041-5A-PCR2
17AL039-10A-PCR2
17AL041-5B-PCR1
17AL039-5B-PCR2
18AL300-5(1)-PCR2
17AL039-5B-PCR1
17AL039-pellet-PCR2
18AL297-5(1)-PCR2
17AL041-10A-PCR2
17AL041-10A-PCR1
17AL039-10B-PCR2
17AL040-5A-PCR1
17AL041-10B-PCR2
18AL297-10-PCR1
17AL095-5(1)-PCR1
18AL320-10(1)-PCR2
19AL419-5(1)-PCR2
19AL419-5(1)-PCR1
18AL320-5(2)-PCR1
18AL320-10(2)-PCR2
18AL320-10(1)-PCR1
18AL297-5(1)-PCR1
18AL320-10(2)-PCR1
17AL040-5B-PCR2
18AL297-10-PCR2
17AL039-pellet-PCR1
18AL297-5(2)-PCR1
17AL039-10A-PCR1
17AL095-10(2)-PCR1
17AL095-10(2)-PCR2
18AL599-5(2)-PCR2
17AL041-5A-PCR1
17AL040-5A-PCR2
18AL320-5(2)-PCR2
18AL300-10-PCR1
18AL300-5(1)-PCR1
17AL095-5(2)-PCR1
17AL286-pellet-PCR1
17AL095-10(1)-PCR2
17AL040-5B-PCR1
17AL095-5(2)-PCR2
17AL041-1-PCR1
18AL300-10-PCR2
17AL041-10B-PCR1
17AL225-10(2)-PCR1
17AL040-10B-PCR1
18AL297-5(2)-PCR2
18AL300-5(2)-PCR2
17AL040-10A-PCR2
17AL040-10A-PCR1
17AL286-pellet-PCR2
17AL095-10(1)-PCR1
18AL300-5(2)-PCR1
17AL040-10B-PCR2
17AL225-1-PCR1
17AL225-1-PCR2

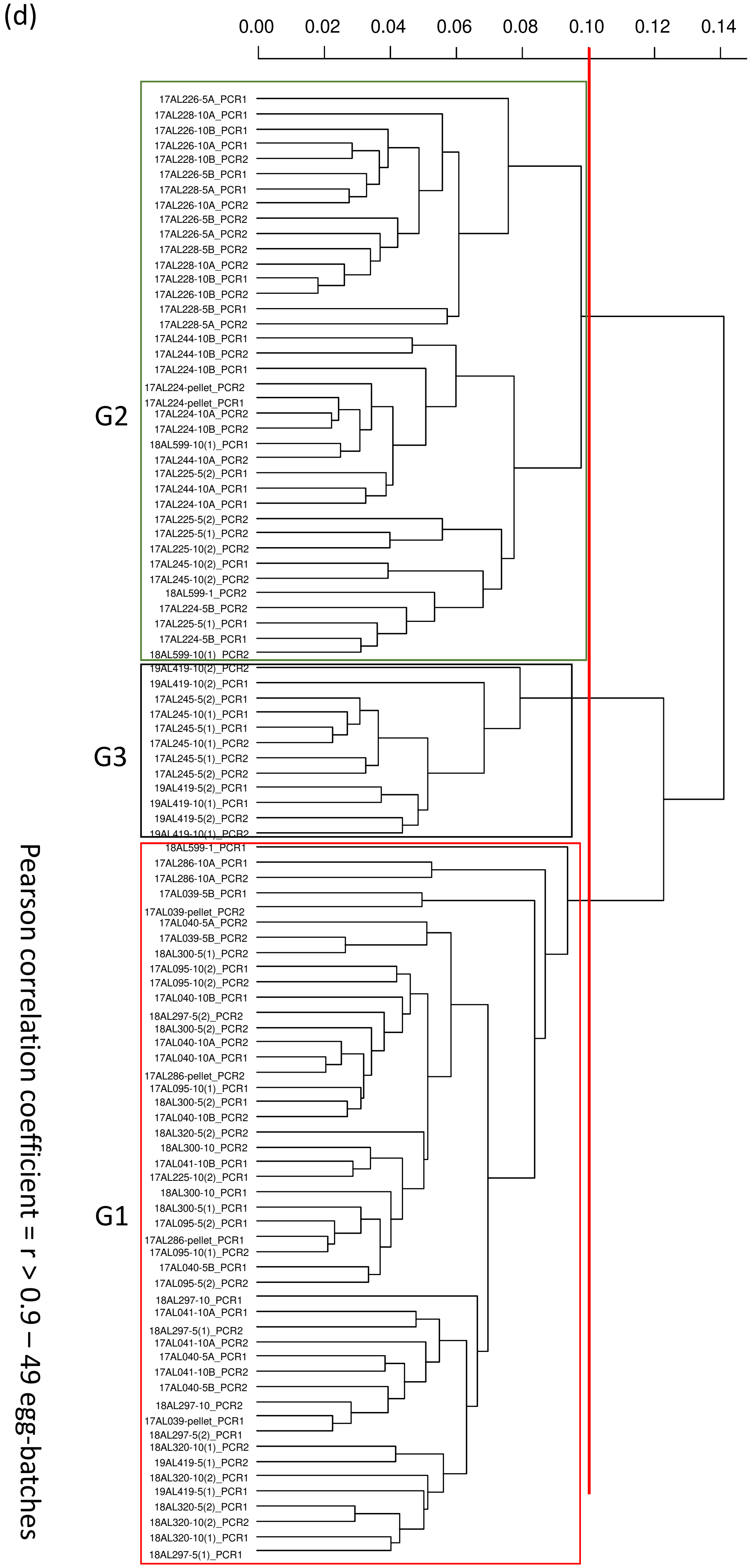
(d)
0.00
0.02
0.04
0.06
0.08
0.10
0.12
0.14
G2
17AL226-5A_PCR1
17AL228-10A_PCR1
17AL226-10B_PCR1
17AL226-10A_PCR1
17AL228-10B_PCR2
17AL226-5B_PCR1
17AL228-5A_PCR1
17AL226-10A_PCR2
17AL226-5B_PCR2
17AL226-5A_PCR2
17AL228-5B_PCR2
17AL228-10A_PCR2
17AL228-10B_PCR1
17AL226-10B_PCR2
17AL228-5B_PCR1
17AL228-5A_PCR2
17AL244-10B_PCR1
17AL244-10B_PCR2
17AL224-10B_PCR1
17AL224-pellet_PCR2
17AL224-pellet_PCR1
17AL224-10A_PCR2
17AL224-10B_PCR2
18AL599-10(1)_PCR1
17AL244-10A_PCR2
17AL225-5(2)_PCR1
17AL244-10A_PCR1
17AL224-10A_PCR1
17AL225-5(2)_PCR2
17AL225-5(1)_PCR2
17AL225-10(2)_PCR2
17AL245-10(2)_PCR1
17AL245-10(2)_PCR2
18AL599-1_PCR2
17AL224-5B_PCR2
17AL225-5(1)_PCR1
17AL224-5B_PCR1
18AL599-10(1)_PCR2
G3
19AL419-10(2)_PCR2
19AL419-10(2)_PCR1
17AL245-5(2)_PCR1
17AL245-10(1)_PCR1
17AL245-5(1)_PCR1
17AL245-10(1)_PCR2
17AL245-5(1)_PCR2
17AL245-5(2)_PCR2
19AL419-5(2)_PCR1
19AL419-10(1)_PCR1
19AL419-5(2)_PCR2
19AL419-10(1)_PCR2
G1
18AL599-1_PCR1
17AL286-10A_PCR1
17AL286-10A_PCR2
17AL039-5B_PCR1
17AL039-pellet_PCR2
17AL040-5A_PCR2
17AL039-5B_PCR2
18AL300-5(1)_PCR2
17AL095-10(2)_PCR1
17AL095-10(2)_PCR2
17AL040-10B_PCR1
18AL297-5(2)_PCR2
18AL300-5(2)_PCR2
17AL040-10A_PCR2
17AL040-10A_PCR1
17AL286-pellet_PCR2
17AL095-10(1)_PCR1
18AL300-5(2)_PCR1
17AL040-10B_PCR2
18AL320-5(2)_PCR2
18AL300-10_PCR2
17AL041-10B_PCR1
17AL225-10(2)_PCR1
18AL300-10_PCR1
18AL300-5(1)_PCR1
17AL095-5(2)_PCR1
17AL286-pellet_PCR1
17AL095-10(1)_PCR2
17AL040-5B_PCR1
17AL095-5(2)_PCR2
18AL297-10_PCR1
17AL041-10A_PCR1
18AL297-5(1)_PCR2
17AL041-10A_PCR2
17AL040-5A_PCR1
17AL041-10B_PCR2
17AL040-5B_PCR2
18AL297-10_PCR2
17AL039-pellet_PCR1
18AL297-5(2)_PCR1
18AL320-10(1)_PCR2
19AL419-5(1)_PCR2
18AL320-10(2)_PCR1
19AL419-5(1)_PCR1
18AL320-5(2)_PCR1
18AL320-10(2)_PCR2
18AL320-10(1)_PCR1
18AL297-5(1)_PCR1
Pearson correlation coefficient = r > 0.9 – 49 egg-batches
